# Knowledge, attitude and practice regarding folic acid deficiency; A hidden hunger

**DOI:** 10.12669/pjms.303.4716

**Published:** 2014

**Authors:** Aliya Hisam, Mahmood Ur Rahman, Syed Fawad Mashhadi

**Affiliations:** 1 Dr. Aliya Hisam, MBBS, MPH, Lecturer, Community Medicine Department, Army Medical College, Rawalpindi, Pakistan.; 2 Mahmood Ur Rahman, MBBS, DPH, MPH, MSc, FCPS, Professor and Head, Community Medicine Department, Army Medical College, Rawalpindi, Pakistan.; 3 Syed Fawad Mashhadi, MBBS, MPH, Lecturer, Community Medicine Department, Army Medical College, Rawalpindi, Pakistan.

**Keywords:** Attitude, Folic acid Deficiency, Knowledge, Practice

## Abstract

***Objectives:*** To find the Knowledge Attitude and Practice regarding Folic Acid Deficiency among Women of Child Bearing Age (WPCBA). To find out the Association of Education Level with Practice of Folic Acid in WPCBA.

***Methods:*** A Descriptive cross sectional study (Knowledge Practice and Attitude) was conducted at Military Hospital and Combined Hospital Rawalpindi from September 2012 to February 2013. About 400 married females of age group 21-42 years were included by convenient sampling technique. After taking informed verbal consent, a closed ended interviewer administered questionnaire was filled. Data was entered and analyzed using SPSS version 20.

***Results:*** Mean age of the respondents was 30.31 + 5.280 years. Illiterate and literate were 165 (41.25%) and 235 (58.75%) respectively. The knowledge regarding folic acid need was 172 (43%). Only 161 (40.25%) thought that folic acid deficiency in pregnant women results in abnormality in newborn. In pregnancy, 205 (51.25%) had received folic acid supplementation. Association between education level and practice of folic acid was significant (p= 0.009) at 95% confidence level.

***Conclusion:*** Knowledge regarding folic acid deficiency among WOCBA was low along with the poor attitude. Practice was also not satisfactory. Education status plays important role in preventing micronutrient deficiency.

## INTRODUCTION

Vitamin B_9_ (folic acid and folate) is essential to numerous bodily functions. The human body needs folate to synthesize, repair, and methylate DNA as well as to act as a cofactor in certain biological reactions. It is especially important in aiding rapid cell division and growth, such as in infancy and pregnancy. Maternal folate deficiency is associated with neural tube defects (NTDs).^1^

Globally, an estimated two billion people are affected by deficiencies of essential vitamins and minerals, collectively known as hidden hunger, which negatively impact on health and economic development. The unified global efforts to mitigate the hidden hunger, in populations around the world are crucial to the achievement of most of the Millennium Development Goals (MDGs). A number of countries in sub-Saharan Africa, as well as India and Afghanistan, had an alarmingly high level of hidden hunger, with stunting, iron deficiency anemia, and vitamin A deficiency all being highly prevalent. In 36 countries, deficiencies of micronutrients were responsible for 1.5-12% of the total DALYs.^2^

According to a research, there is significant dose--response relationship between folate intake and birth weight. This relationship indicated 2% increase in birth weight for every two-fold increase in folate intake.^3^ Less folic intake during pregnancy may be associated with autism in newborn infants.^4^ Preliminary research has shown a possible link between folic acid and Down syndrome.^5^ Folic acid supplements consumed before and during pregnancy may reduce risk of heart defects in infants.^6^

The worldwide incidence of neural tube defects (NTDs) ranges from 1.0 to 10.0 per 1,000 births with almost equal frequencies between two major categories: anencephaly and spina bifida (SB).^7^ In USA, the numbers of annual NTD-affected birth defects were calculated from a 24-month pre-fortification period (1995-1996) and a 24-month post-fortification period (1999-2000).^8^ An estimated 2,490 spina bifida--affected pregnancies and 1,640 anencephaly affected pregnancies occurred annually before fortification of food with folic acid, after fortification, an estimated 1,640 spina bifida--affected pregnancies and 1,380 anencephaly-affected pregnancies occurred, for an annual average of 3,020 NTD-affected pregnancies (a 27% decline). According to a study done in Swat (Pakistan) prevalence of anencephaly was 11.33/1000 births and that of spina bifida was 0.72/1000 births.^9^ According to a survey the pregnant anemic women visiting tertiary care hospital of Rawalpindi had iron (57%); folate (20%), followed by combined iron folate (19%) and cobalamine (4%) deficiency during first antenatal visit.^10^

Adequate folate intake during the preconception period (which is the time right before and just after a woman becomes pregnant) helps protect against a number of congenital malformations, including neural tube defects (which are the most notable birth defects that occur from folate deficiency).^11^

Folic acid supplementation has not been shown to reduce risk of cardiovascular diseases or all-cause mortality among participants with prior history of vascular disease^12^ but its effects during pre-pregnancy and first trimester in reducing incidence of several abnormalities in newborn is clearly established in different studies.^1^^,^^3^^,^^4^

As we are progressing towards the Millennium Development Goals (MDGs), it is essential to identify the burden of disease of micronutrient deficiency. It was essential to first assess the KAP (i.e. sources, benefits, requirement in pregnancy, use of folic acid supplements, folic acid intake in pregnancy) of this important micronutrient i.e. folic acid and then spread the word regarding its importance in pre-conception and antenatal period as to prevent the most preventable disease i.e. newborn abnormality. The necessity of this study was to create awareness in community regarding the hidden hunger by creating awareness regarding importance of this micronutrient deficiency (folic acid) especially in the most vulnerable group that is women of child bearing age (21-42 years of age) visiting tertiary care hospital in Rawalpindi. Association between the education level and practice of folic acid intake was also analyzed.

## METHODS

A descriptive cross sectional (Knowledge Practice and Attitude) study was conducted in Military Hospital and Combined Military Hospital in Rawalpindi from September 2012 to February 2013. Using WHO sample size calculator, 400 sample size was calculated with confidence level of 95%, anticipated population proportion (p) of 0.2 and absolute precision (d) of 0.04. Non-probability convenient sampling technique was used. Patients were selected on the basis of inclusion criteria that is married females of 21 to 42 years of age. Un-married women and married women having fertility problems or any history of gynecological problem like fibroids, menorrhagia, amenorrhoea and Pelvic Inflammatory disease were excluded from the study. Tool was closed ended questionnaire. Patients coming to outpatient department, emergency and different wards of Military Hospital and Combined Military Hospital of Rawalpindi were selected. Permission from the hospital ethical committee was taken. From the patients informed verbal consent was taken. Brief history was taken and general physical examination was also done.

Data was entered and analysed using Statistical package for Social Sciences (SPSS) version 20. Data including variable such as age, education, knowledge, attitude and practice were presented in the form of frequencies and percentages. Chi-square test of significance with 95% confidence level was used to find association between education level and practice of folic acid intake among WOCBA.

## RESULTS

**Fig.1:**
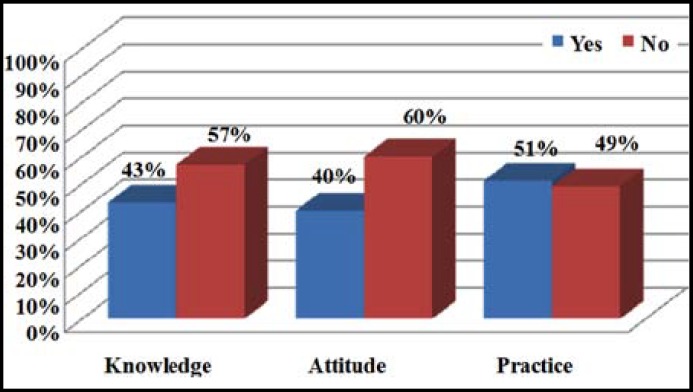
KAP of folic acid deficiency among women of child bearing age (n=400).

**Fig.2:**
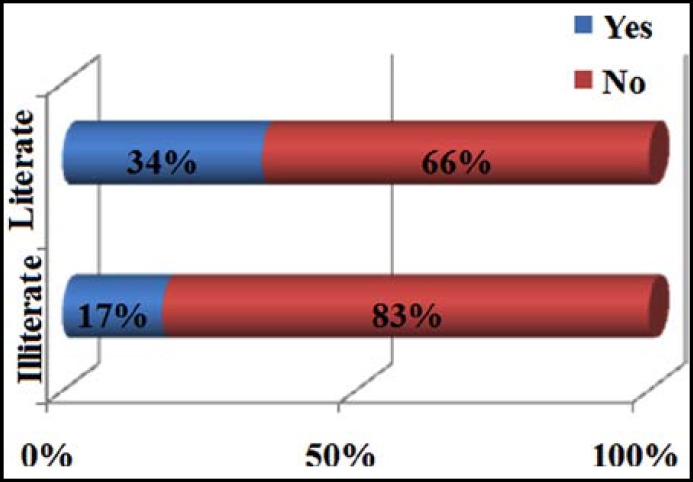
Significant association of education level with practice of folic acid (p=0.009).

Mean age of the respondent was 30.31 + 5.280 years. Illiterate and literate were 165 (41.25%) and 235 (58.75%) respectively. About 215 (53.75%) individuals had two or less than two children while 185 (46.25%) had more than two children. 261 (65.25%) did not had any history regarding abortion or still birth while 139 (34.75%) had positive history. Remarkably 309 (77.25%) had no history of low birth weight babies while only 91(22.75%) had positive history of low birth weight. Regarding birth to child with defects, 42 (10.5%) had positive and 358 (89.5%) had negative history. 219 (54.75%) were informed by gynecologist/physician regarding the importance of folic acid while 181 (45.25%) were not. There were 108 (27.0%) who were informed by parents, friends or relatives regarding folic acid importance while 292 (73.0%) were not. Media contributed only 64 (16%) awareness regarding folic acid while 336 (83%) were not communicated by media. Regarding pre-pregnancy need of folic acid only 172 (43%) had while 228 (57%) did not had any knowledge regarding its pre-pregnancy importance, shown in Fig.1. 213 (53.25%) knew that during the first three months folic acid intake is important while 187 (46.75%) did not know this fact. Only 161 (40.25%) thought that folic acid deficiency among pregnant women results in abnormality in new born while 239 (59.75%) didn’t knew regarding its importance. Total 237 (60.25%) had idea about the rich source of folic acid but 163 (40.75%) didn’t knew regarding its source. Only 93 (23.25%) were regularly having green vegetables and fruits and 307 (76.75%) were not having green leafy vegetables and fruits in their diet regularly. Only 205 (51.25%) had received folic acid supplementation during pregnancy while 195 (48.75%) had not received any supplementation. About 209 (52.25%) participants knew that nutritional value of food decline with excessive cooking but 191 (47.75%) didn’t knew this fact. 23 (5.75%) thought that folic acid is harmful for health but 377 (94.25%) did not. 128 (32%) thought birth defect due folic acid deficiency is a hidden hunger and an emerging health problem in our society and 273 (68%) though it is not an emerging health problem, details in Table-I and Fig.1. One hundred and thirty eight (34.5%) wanted to attend educational lectures or workshop on folic acid supplementation and 262 (65.5%) did not wanted to attend such workshops. Significant association of literacy level and practice of folic acid intake during women of child bearing age was found (p=0.009) at 95% confidence level, as shown in Fig.2.

**Table-I:** KAP of Folic Acid among Women of Child Bearing Age (WOCBA) (N= 400).

*Variables*	*Frequencies (%)*	
Age ± Standard Deviation	30.31 + 5.280 years	
Education Status: Illiterate Literate	165 (41.25%) 235 (58.75%)	(p = 0.009)
Do you have:	Yes	No
History of Low Birth Weight:	91 (22.75%) 309 (77.25%)	
Birth to child with Defects:	42 (10.5%) 358 (89.5%)	
From where did you received awareness of folic acid: Gynaecologist/Physician Parents/Friends/Relatives Media	219 (54.75%) 108 (27%) 64(16%)	181 (45.25%) 292 (73%) 336 (83%)
Knowledge: In pre-pregnancy, there is a need of folic acid supplementation? Is first trimester Folic acid intake important Do you know about Folic acid rich food sources Does excessive cooking declines food nutritional value	172 (43%) 213 (53.25%) 237 (60.25%) 209 (52.25%)	228 (57%) 187 (46.75%) 163 (40.75%) 191(47.75%)
Attitude: Will folic acid deficiency leads to abnormality in new born Are you willing to know more about folic acid	161 (40.25%) 138 (34.5%)	239 (59.75%) 262(65.5%)
Practice: Did you took folic acid supplements in pregnancy Do you have intake of folic acid rich foods in your diet	205 (51.25%) 93 (23.25%)	195 (48.75%) 307 (76.75%)
Is Folic Acid Deficiency a Hidden Hunger and an Emerging Health Problem in our society?	128 (32%) 273 (68%)	

## DISCUSSION

This descriptive cross sectional (knowledge, attitude and practice) study regarding folic acid deficiency among women of child bearing age concluded that there is a wide deficiency in knowledge, attitude and practice regarding this important micronutrient in the most vulnerable group that is women of child bearing age. Knowledge regarding the need for folic acid in pregnancy was 172 (43%). Varied attitude was observed as 161 (40.25%) thought that folic acid deficiency in pregnancy result in newborn abnormality and only 172 (34.5%) were interested in attending the awareness campaign regarding folic acid importance. Practice was also poor as only 172 (43%) were regularly having folic acid rich foods while 205(51.25%) had received folic acid supplementation during pregnancy. This study highlighted that there is a continueous need to educate and motivate women of child bearing age regarding this hidden hunger, to motivate the most vulnerable group regarding the use of this micronutrient before conception and during 1^st^ trimester of pregnancy to avoid congenital defects in newborns of children. Through this research, it was also established that the literate women had more knowledge about the importance of folic acid and were also having good practice as compared to illiterate women (p>0.009 with 95% CL).

It is widely accepted that pre-conceptional supplementation with folic acid can prevent a significant proportion of neural tube defects (NTDs).^1^^,^^3^^,^^4^ A literature search was conducted to identify studies that included assessment of folic acid knowledge and use in U.K. women of different ethnicities study. It concluded that implementing targeted, innovative education campaigns together with a mandatory fortification policy, including the fortification of ethnic minority foods, will be required for maximum prevention of folic acid-preventable Neural Tube Defects.^13^ Our study targeted WOCBA and identified that there is a great need to spread the message regarding this hidden hunger.

Neural tube defects are reduces by taking folic acid supplements in early pregnancy. Health surveys on knowledge, attitudes and intake of folic acid are limited. A study in China was conducted regarding knowledge and attitude towards practice of taking folic acid and factors affecting the recommendation and prescription among health care professionals. It concluded that although participants had a good knowledge about neural tube defect and folic acid but lack of some knowledge possibly led to a relatively low rate of correct behavior among health care personals. They also recommended to educate health care personals in this regard.^14^ Our study concludes at creating awareness in the community to increase intake of folic acid among WOCBA. It did not explored the factors affecting health care personals prescription rate but it would be good to do a survey regarding factors affecting prescription.

Another study regarding the community pharmacist was conducted to determine the knowledge of folic acid use for neural tube defects prevention. Of the 122 pharmacists who completed the survey, 116 (95.1%) knew that folic acid prevents some birth defects. Twenty-eight (22.9%) responded that they "always" or "usually" discuss multivitamins with women of childbearing potential, and 19 (15.6%) responded that they "always" or "usually" discuss folic acid supplements. Respondents identified continuing education programs, pharmacy journals/magazines, and the Internet as preferred avenues to obtain additional information about folic acid and NTD. The study concluded that community pharmacists can fulfill a vital public health role by counselling women of childbearing potential about folic acid intake. Educational materials may be beneficial in augmenting knowledge of folic acid and facilitating patient education.^15^ In our setup, as there is a wide gap in achieving adequate literacy level, it is quite challenging to think of recommending journals or internet as a source of knowledge for illiterate? We need to first increase the literacy level along with continued awareness campaigns, mass education, health care professionals including pharmacist and especially involving media in communicating the message of importance of folic acid in pregnancy as well as pregnancy with respect to preventing abnormality in new born.

In Japan, a ten year study was conducted to examine whether their life styles have been shifting to the direction of lowering the incidence of spina bifida. Food records were asked from the participants to semi-quantitatively describe diets and beverages they consumed for a 3-day period. Life style questionnaires demonstrated that knowledge of folic acid and the proportion of those who took folic acid supplements elevated from 15.3% and 9.1% in 2002 to 43.7 and 61.5% in 2011, respectively. Three-day food records revealed that the mean dietary folate intakes ranged from 260 to 360 microgm/day in each year which were less than the recommended dietary allowance (RDA) publicized by the government, but that the proportion of pregnant women in the first trimester who consumed folic acid supplements from 4 weeks prior to 12 weeks after conception increased from 7.4% in 2003 to 69.6% in 2011. They concluded that life styles of pregnant women have been shifting toward the direction in the past 10 years where the risk for having a pregnancy afflicted with spina bifida is to be decreased. Medical doctors, nurses, midwives, dieticians and pharmacists are asked to repeatedly supply important information on folic acid and to advise taking folic acid supplements 400 microgram a day to women planning to conceive or women in the reproductive age.^16^ We really need to explore that in what doses, with what frequency, with what continuity and by whom folic acid is to be prescribed.

In another study, Pregestational supplementation of folate prevents neural tube defects and a daily supplemental dose of 400 μg/day of folate is recommended when planning pregnancy.^17^ Preterm infants receiving parenteral nutrition with high folic acid content have no risk of folate deficiency during the two months of age which furthers emphasizes the importance of folic acid in maternal nutrition.^18^

It is widely accepted that adequate maternal consumption of folic acid before pregnancy and during the early weeks of gestation can reduce the risk of having a child with a neural tube defect (NTD). As a result, public health authorities worldwide have recommended consuming 400 μg folic acid per day during the pre-conceptional period to reduce the risk of first occurrence NTDs. This is of particular importance in light of recent speculations that a similar increase in folic acid intake may result in the prevention of most folate-related NTDs.^19^ As Knowledge of FAS is very low among women of childbearing age^20^ if we can impart health education through gynecologists, physicians, media, symposiums and workshops and can enhance the fortification of food with folic acid through government interventions, we can reduce the incidence of neural tube defects and other abnormalities in newborn to quite an extent.

## CONCLUSION

Knowledge regarding folic acid deficiency among WOCBA was low along with the poor attitude. Practice was also not satisfactory as very few had received folic acid supplementation during their pre-pregnancy and antenatal period. Education status plays important role in preventing micronutrient deficiency. There is a dire need to increase awareness regarding importance of this hidden hunger among WOCBA especially by involving health care professionals, community health workers, pharmacist, media, symposiums and workshops as it is completely preventable hidden public health problem. 
